# Surgical Care Required for Populations Affected by Climate-related Natural Disasters: A Global Estimation

**DOI:** 10.1371/currents.dis.e601960a8cd66c3083d160877abfdde4

**Published:** 2016-08-10

**Authors:** Eugenia E. Lee, Barclay Stewart, Yuanting A. Zha, Thomas A. Groen, Frederick M. Burkle, Adam L. Kushner

**Affiliations:** Department of Surgery, University of Southern California, Los Angeles, California, USA; Department of International Health, Johns Hopkins Bloomberg School of Public Health, Baltimore, Maryland, USA; Department of Surgery, University of Washington, Seattle, Washington, USA; School of Medical Sciences, Kwame Nkrumah University, Kumasi, Ghana; Department of Interdisciplinary Health Sciences, Stellenbosch University, Cape Town, South Africa; University of California, Irvine School of Medicine, Irvine, California, USA; Department of International Health, Johns Hopkins Bloomberg School of Public Health, Baltimore, Maryland, USA; Department of Natural Resources, Universiteit Twente, Enschede, the Netherlands; Harvard Humanitarian Initiative, Harvard University, Cambridge, Massachusetts, USA; Surgeons OverSeas (SOS), New York, New York, USA; Department of International Health, Johns Hopkins Bloomberg School of Public Health, Baltimore, Maryland, USA; Department of Surgery, Columbia University, New York, New York, USA

## Abstract

Background: Climate extremes will increase the frequency and severity of natural disasters worldwide.  Climate-related natural disasters were anticipated to affect 375 million people in 2015, more than 50% greater than the yearly average in the previous decade. To inform surgical assistance preparedness, we estimated the number of surgical procedures needed.

Methods: The numbers of people affected by climate-related disasters from 2004 to 2014 were obtained from the Centre for Research of the Epidemiology of Disasters database. Using 5,000 procedures per 100,000 persons as the minimum, baseline estimates were calculated. A linear regression of the number of surgical procedures performed annually and the estimated number of surgical procedures required for climate-related natural disasters was performed.

Results: Approximately 140 million people were affected by climate-related natural disasters annually requiring 7.0 million surgical procedures. The greatest need for surgical care was in the People’s Republic of China, India, and the Philippines. Linear regression demonstrated a poor relationship between national surgical capacity and estimated need for surgical care resulting from natural disaster, but countries with the least surgical capacity will have the greatest need for surgical care for persons affected by climate-related natural disasters.

Conclusion: As climate extremes increase the frequency and severity of natural disasters, millions will need surgical care beyond baseline needs. Countries with insufficient surgical capacity will have the most need for surgical care for persons affected by climate-related natural disasters. Estimates of surgical are particularly important for countries least equipped to meet surgical care demands given critical human and physical resource deficiencies.

## Introduction

Climate extremes are expected to increase the number and severity of natural disasters worldwide.^1^ Climate-related natural disasters were anticipated to affect 375 million people in 2015, which is more than 50% greater than the yearly average in the previous decade.^2^ To inform national and humanitarian surgical assistance preparedness, we estimated the number of surgical procedures needed by this population in 2016.

## Methods

Data on the numbers of people affected by climate-related natural disasters (e.g. earthquakes, dry mass movements, floods, landslides, severe storms) from 2004 to 2014 were obtained from the Centre for Research of the Epidemiology of Disasters (CRED) database.^3^ Using 5,000 procedures per 100,000 persons as the minimum number of procedures that a population requires,^4^ baseline estimates were calculated for affected populations. Additionally, the estimated number of persons who sustained an injury that was related to each disaster was obtained from the CRED database. The number of injuries was used to calculate additional surgical needs assuming that most injuries, at minimum, required a surgical evaluation. Lastly, we performed a linear regression of the estimated number of surgical procedures performed per year and the estimated number of surgical procedures required for climate-related natural disasters. By doing so, the current global preparedness to meet the potential surgical demand from climate-related natural disasters could be described.

## Results

On average, 140 million people were affected by climate-related natural disasters annually (range 69 – 205 million people annually). Therefore, at least 7.0 million surgical procedures were required to care for this population. Additionally, an estimated 138,781 persons were injured by climate-related natural disasters each year. Assuming all injuries in these settings required a surgical evaluation, surgical need increased by 2.0% from the needs of a population unaffected by disaster (138,781 additional surgical evaluations and/or procedures per year). The greatest need for surgical care was in the People’s Republic of China, India, and the Philippines with approximately 4.0, 0.8 and 0.5 million procedures, respectively (Figure 1). The countries with the highest percentage of the population affected were St Lucia (11% of the population), the Philippines (10%), and the People’s Republic of China (5.9%). The estimated rate of surgical procedures in 2008 based on per-capita expenditure in these countries was 2,578 (St Lucia), 2,168 (Philippines), and 2,659 (China) per 100,000 population, all significantly below the baseline goal of 5,000 procedures per 100,000 population.^5^ Linear regression demonstrated a poor correlation with current national surgical capacity, as measured by the rate of surgical procedures performed per 100,000 population per year, and the estimated procedures required for persons affected by climate-related natural disasters (Figure 2; -3.1252x + 56295; R2=0.0014). In other words, there seems to be a poor relationship between national surgical capacity and estimated need for surgical care resulting from natural disaster. However, it appears that countries with the least surgical capacity will have the greatest need for surgical care for persons affected by climate-related natural disasters.

**Minimum Surgical Procedures Resulting from Natural Disasters Needed:**
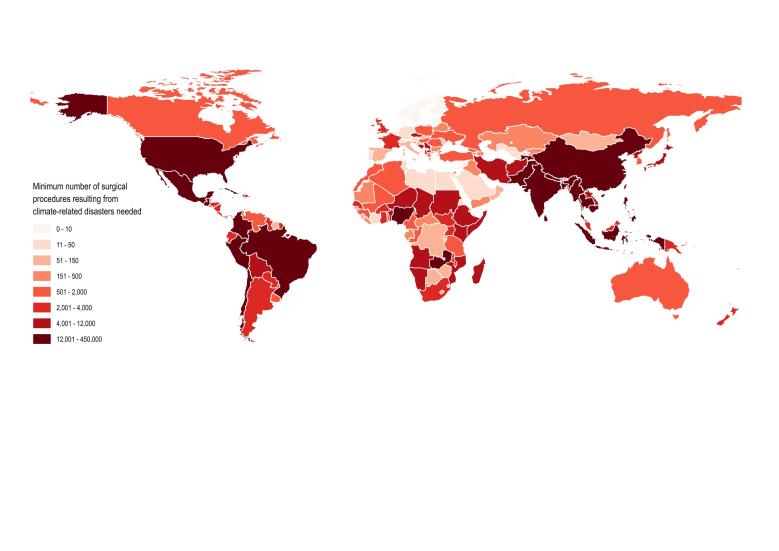

**Annual procedures needed versus performed:**
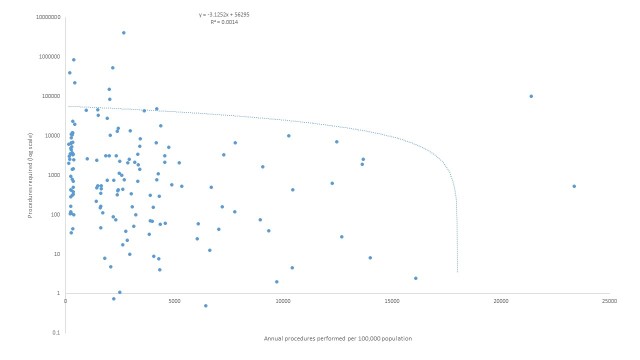

## Discussion

As climate extremes increase the frequency and severity of natural disasters, hundreds of millions of people will be affected and millions will need surgical care beyond baseline needs. Currently, countries with insufficient surgical capacity are expected to have the most need for surgical care for persons affected by climate-related natural disasters. Estimates of surgical need are important as they inform national policy, humanitarian assistance programs, and implementing partners, as well as support surgical advocacy. Such estimates are particularly important for low- and middle-income countries, which are least equipped to meet surgical care demands given critical human and physical resource deficiencies.^6^

Currently, it is difficult to contextualize our findings as other published data on this topic do not exist. However, an example from a natural disaster demonstrates the importance of estimating potential surgical need and preparing humanitarian and national surgical capacity. In April and May of 2015, the Nepal earthquakes directly affected 5.6 million people^3^ whose surgical needs included an estimated 281,605 procedures. However, the entire natural surgical capacity is less than 100,000 procedures per year (95% confidence interval 80,532 to 96,432 operations annually).^5^ Furthermore, healthcare infrastructure was destroyed by the earthquakes, which crippled the local capability to meet surgical needs. This example demonstrates that without dedicated efforts to increase humanitarian surgical preparedness globally and national capacity in vulnerable nations, victims of future disasters will encounter similar deficiencies.

While these estimates highlight the need to plan for surgical care provision during climate-related disaster, there are limitations. First, the number of injured persons that needed care during a natural disaster from the CRED database is likely an underestimate as many victims received care outside of the disaster zones or were not recorded. Thus, these results are bare minimum estimates of surgical need. Second, data on the number of procedures provided by national or international organizations during or in the aftermath of disasters were not available. Thus, we were not able determine the surgical need that was relieved by local healthcare systems or humanitarian assistance programs. Lastly, these estimates represent average surgical needs. Disasters both sudden onset and slow-moving cause variable numbers of injuries and degrees of disruption in sanitation and destruction of local healthcare infrastructure. Therefore, plans for preparing surgical care in case of disaster should consider best and worst case scenarios.

While no countries are immune, climate-related changes are increasingly influencing migration, both externally and internally, in the poorest and most vulnerable populations worldwide. Environmental migration is expensive; the wealthier, including physicians and other healthcare providers, are more able to relocate disrupting the indigenous health system capacity even further.^7^ Migrants entering the European Union do so for a variety of reasons. Increasing numbers escaping natural disasters, environmental and economic fears and conflict seek immediate healthcare in the countries they enter. In one study of 880 asylum seekers presenting at a Central European emergency department, 43.3% did so for surgical problems, the most common being trauma-related (50.6%).^8^ In the developed world, climatic factors in Australia has caused the largest depletion of stratospheric ozone resulting in major increases in melanomas, rare skin cancers and cataracts requiring both medical and surgical interventions.^9^ No comparable studies exist for the developing world.

At baseline, only 15% of the world’s countries are able to provide a minimum of 5,000 procedures per 100,000 persons.^4^ Countries most at risk of climate-related natural disasters frequently have among the lowest procedure rates. Investment in surgical care in countries with particularly low capacity and high natural disaster risk is urgently needed to strengthen their resilience should a disaster occur. Communities must also provide social and welfare services which are uniquely influenced by both the resources available and the individual differences of cultural identity and health beliefs.^10^ Given the potential for healthcare infrastructure destruction from a natural disaster, humanitarian surgical assistance must also be readied to meet the surgical needs of those affected.

## Competing Interests

The authors have declared that no competing interests exist

## Data Availability

The data set used for this research was obtained from the Centre for Research of the Epidemiology of Disasters (CRED) International Disaster Database: http://www.emdat.be/

## Correspondence

Eugenia E. Lee, MD, MPH

E-mail: eugenialee@jhu.edu
